# Comprehensive analysis of epigenomics and transcriptome data to identify potential target genes associated with obesity

**DOI:** 10.3389/fgene.2022.1024300

**Published:** 2022-10-14

**Authors:** Peili Wu, Lei Guo, Xuelin Li, Yuejun Du, Xiaochun Lin, Xiaoqin Ma, Yingbei Lin, Churan Wen, Chuyi Yang, Nannan Liu, Qijian Feng, Yaoming Xue, Meiping Guan

**Affiliations:** ^1^ Department of Endocrinology and Metabolism, Nanfang Hospital, Southern Medical University, Guangzhou, Guangdong, China; ^2^ Department of Urology, Nanfang Hospital, Southern Medical University, Guangzhou, Guangdong, China

**Keywords:** obesity, methylation, immune infiltration, metabolism, comprehensive analysis

## Abstract

DNA methylation is closely related to the occurrence and development of many diseases, but its role in obesity is still unclear. This study aimed to find the potential differentially methylated genes associated with obesity occurrence and development. By combining methylation and transcriptome analysis, we identified the key genes in adipose tissue affecting the occurrence and development of obesity and revealed the possible molecular mechanisms involved in obesity pathogenesis. We first screened 14 methylation-related differential genes and verified their expression in adipose tissue by quantitative polymerase chain reaction (qPCR). Seven genes with the same expression pattern were identified as key genes, namely, *CCRL2*, *GPT*, *LGALS12*, *PC*, *SLC27A2*, *SLC4A4*, and *TTC36*. Then, the immune microenvironment of adipose tissue was quantified by CIBERSORT, and we found that the content of M0 macrophages and T follicular helper cells in adipose tissue was significantly increased and decreased, respectively, in the obese group. Furthermore, the relationship between key genes and the immune microenvironment was analyzed. Additionally, the metabolic pathway activity of each sample was calculated based on the ssGSEA algorithm, and the key gene–metabolic network was constructed. Moreover, we performed a CMAP analysis based on the differential genes in adipose tissue to screen out drugs potentially effective in obesity treatment. In conclusion, we identified seven methylation-related key genes closely related to obesity pathogenesis and explored the potential mechanism of their role in obesity. This study provided novel insights into the molecular mechanisms and management of obesity.

## Introduction

Obesity is a chronic metabolic disease. Currently, the incidence rate of obesity and obesity-based metabolic diseases is increasing worldwide (Ng et al., 2014), greatly reducing the quality of life. Therefore, it is increasingly important to explore and develop effective treatments for obesity. Obesity is caused by excessive accumulation and abnormal distribution of body fat due to long-term energy intake exceeding consumption. Adipose tissue is the core of the bodys’ energy balance and metabolic homeostasis. Targeting adipose tissue metabolic activity is one of the most attractive strategies to effectively reduce weight and improve metabolism.

Adipose tissue is mainly divided into two categories, brown adipose tissue (BAT) and white adipose tissue (WAT). Among them, BAT resists cold and increases energy consumption by increasing heat production, while WAT, as the main site for nutrition storage, secretes important endocrine hormones to communicate with the central nervous system and other peripheral tissues, which jointly maintain the changing nutritional environment (Kajimura et al., 2015; Chen et al., 2017). Additionally, WAT can also coordinate with immune cells and play an important role in integrating immune and metabolic signals (Martinez-Santibanez and Lumeng, 2014; Crewe et al., 2017), revealing a potential new target for preventing and treating obesity and its related complications by regulating the immune microenvironment.

DNA methylation is associated with causing genetic changes without changing the DNA sequence, which is one of the most stable epigenetic mechanisms. DNA methylation is widely involved in the occurrence and development of various characteristics and diseases. Methylation of DNA is generally associated with elevated gene expression, whereas unmethylated DNA is associated with repressed gene expression (Parle-McDermott and Ozaki, 2011). The mechanism is believed to be linked with transcriptional silencing involving the interference of RNA polymerase complex and associated transcription factors (Klose and Bird, 2006; Parle-McDermott and Ozaki, 2011). In obesity, studies have shown that DNA methylation can regulate the homeostasis of energy balance in WAT. Previous studies have reported that methylation changes of certain genes, such as ATP binding cassette subfamily G member 1 (*ABCG1*), sterol regulatory element binding transcription factor (*SREBP1*), and carnitine palmitoyl transferase 1A (*CPT1A*), were related to obesity, and these DNA methylation-related markers might act as predictors of obesity and related diseases. However, due to the unsatisfactory duplication or validation results of DNA methylation markers in obesity, clinical application of DNA methylation-related genes is still relatively limited. Hence, further studies on the key genes of DNA methylation are needed in the future to lay a foundation for them as predictors of obesity and related diseases.

Differential genes and methylation profiles between the control and obese groups were analyzed by combining methylation and transcriptome data to seek potential differentially methylated genes associated with obesity. Furthermore, the selected candidate genes were subsequently verified in human adipose tissue samples. The immune microenvironment and metabolic regulation pathway were further explored, and the relationship between them and the key genes was also analyzed. Additionally, drug prediction was performed based on differential genes of adipose tissue, and potential small molecules for obesity treatment were screened. This study provided potential obesity-related therapeutic targets for further research and obesity management in clinical practice.

## Materials and methods

### Data acquisition

The GEO database (https://www.ncbi.nlm.nih.gov/geo) stores gene expression data from all over the world (Barrett et al., 2007). In this study, three datasets based on subcutaneous adipose analysis were enrolled. The 450-k methylation matrix within GSE67024 (GPL13534) was downloaded from the GEO database, including 15 obese patients (BMI 41.36 ± 4.54 kg/m^2^) and 14 never-obese control women (BMI 25.11 ± 2.49 kg/m^2^). The expression profile of GSE174475 (GPL18573) was downloaded, including 14 obese patients (BMI 34.31 ± 3.17 kg/m^2^) and 29 non-obese control women (BMI 26.45 ± 2.47 kg/m^2^). The expression profile of GSE156906 (GPL24676) was acquired, including 52 obese patients with an average BMI near 38.45 kg/m^2^ and 14 non-obese control with an average BMI of 22.90 kg/m^2^ (Fuchs et al., 2021). Age and gender between obese and non-obese patients in the datasets show no statistical difference (more details in Supplementary Tables S1–S3.

### Differential gene expression analysis and enrichment analysis

The limma package is a commonly used method to identify differential genes (Ritchie et al., 2015). In this study, the R package “limma” was used to analyze the differentially expressed genes between control and obese samples. Filter criteria of differentially expressed genes were set as | log2 fold change | of >1 and a p of <0.05. Differential gene volcano plots were drawn using the R package “ggplot2” (https://link.springer.com/book/10.1007/978-3-319-24277-4), and differential gene heatmaps were drawn using the “pheatmap” package. The “clusterProfiler” (Yu et al., 2012) package was used to perform GO and KEGG pathway enrichment analysis, and the possible pathways of differential genes involved in the progression of obesity were comprehensively explored. GO analysis included three different categories, namely, biological process (BP), molecular function (MF), and cellular component (CC). Enriched pathways with both P and Q values less than 0.05 were considered to be significant.

### DNA methylation analysis

ChAMP is a comprehensive methylation analysis pipeline including functions of low-quality probe filtering, differential methylation position detection, methylated genomic block detection, and so on. In this study, the methylation data in the GSE67024 dataset were analyzed by the R package “ChAMP” (Morris et al., 2014; Tian et al., 2017), and the methylation levels of adipose tissue genes between the control and the obese groups were compared. The differentially methylated genes were screened. Filter criteria of differentially methylated genes were |log2 fold change| of >0.2 and an adjusted P of <0.05. Lower-expression genes with hypermethylation and higher-expression genes with hypomethylation were identified as key genes in disease development.

### Patients and quantitative real-time PCR

Patients who met the inclusion criteria were enrolled: a. aged 18–65; b. subcutaneous adipose tissue and clinical data available; c. informed consent. Subcutaneous adipose tissue was obtained from 14 men and 10 women with diseases such as renal cysts, kidney stones, adrenal adenoma, and primary aldosteronism, who underwent laparoscopic adrenalectomy (more details in Supplementary Materials). According to the Chinese standard of obesity, in this study, the obese group was set with a BMI of no less than 28 kg/m^2^. The average BMI in the obese group was significantly different compared to that of the control group (30.01 ± 1.48 kg/m^2^ vs. 22.43 ± 2.81 kg/m^2^, *p*＜0.001). No significant difference in age and gender between the two groups was observed (more details in Supplementary Table S4. It was approved by the Ethics Committee of Nanfang Hospital, and all subjects. Total RNA was extracted from subcutaneous adipose tissue, cDNA was reverse-transcribed, and quantitative reverse transcription-polymerase chain reaction (RT-PCR) was conducted on the QuantStudio5 real-time PCR system. All primers were confirmed by PubMed BLAST, and human β-ACTIN served as the internal control. Primer sequences are given in the Supplementary Materials.

### Immune cell infiltration

CIBERSORT is a deconvolution method that effectively links the gene expression data with the content of immune cells and quantifies the composition of immune cells through gene expression profiles (Chen et al., 2018). For each case, the total of predicted cell subgroups was equal to 1, which indicates the enrichment of immune cell infiltration. This study quantified the immune microenvironment of adipose tissue gene expression data of GSE174475 by the CIBERSORT algorithm, inferred the relative content of immune cells in adipose tissue, and compared the relative level of immune cell content in different groups. Additionally, Pearson’s correlation analysis was used to explore the correlation between key genes and the content of immune cells, and *p* < 0.05 was considered statistically significant.

### Connectivity map analysis of drugs

The connectivity map (CMap) is a database for drug prediction based on differential gene expression (Lamb et al., 2006). It contains 6100 examples of 1309 small-molecule drugs and is mainly used to reveal the functional relationship between genes, drugs, and diseases. In the present study, we predicted potential molecular compounds for obesity treatment based on the CMAP database. We first analyzed the differential genes between the control and obese groups in adipose tissue and then input the differential genes into the CMAP database for drug prediction. The correlation between drugs and differential genes was displayed by a score from −1 to 1, with a negative number indicating the gene expression pattern of the corresponding interference, suggesting that this interference might have a potential therapeutic effect on obesity.

### Statistical analysis

Data analysis was conducted by the R framework (version 4.1.2). All statistical tests were bilateral, and a *p*-value < 0.05 was considered statistically significant.

## Results

### Differential expression analysis of expression profiles and methylation, gene ontology, and Kyoto Encyclopedia of Genes and Genomes Enrichment Analysis

**FIGURE 1:**
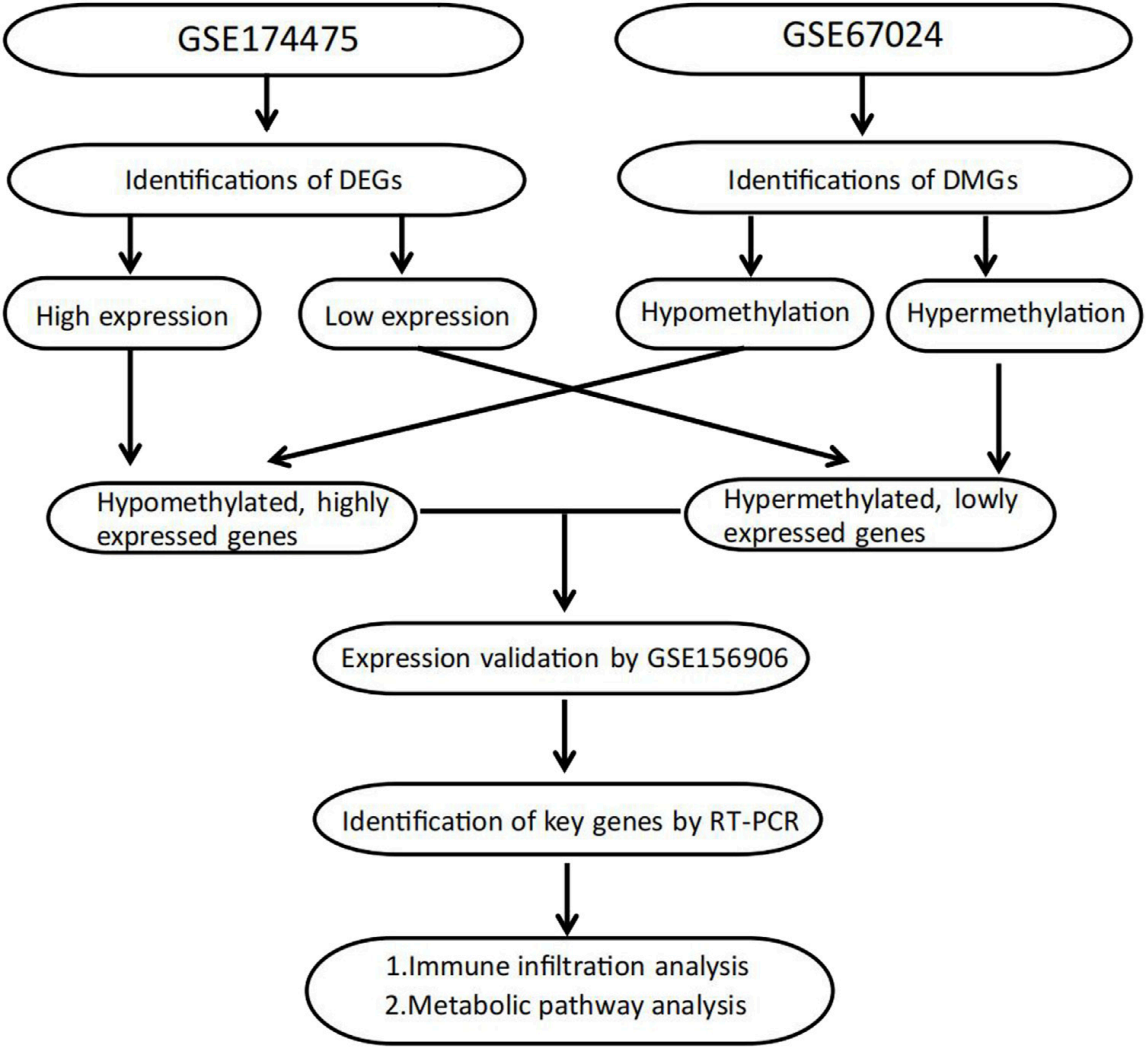
Flowchart of study design.

**FIGURE 2:**
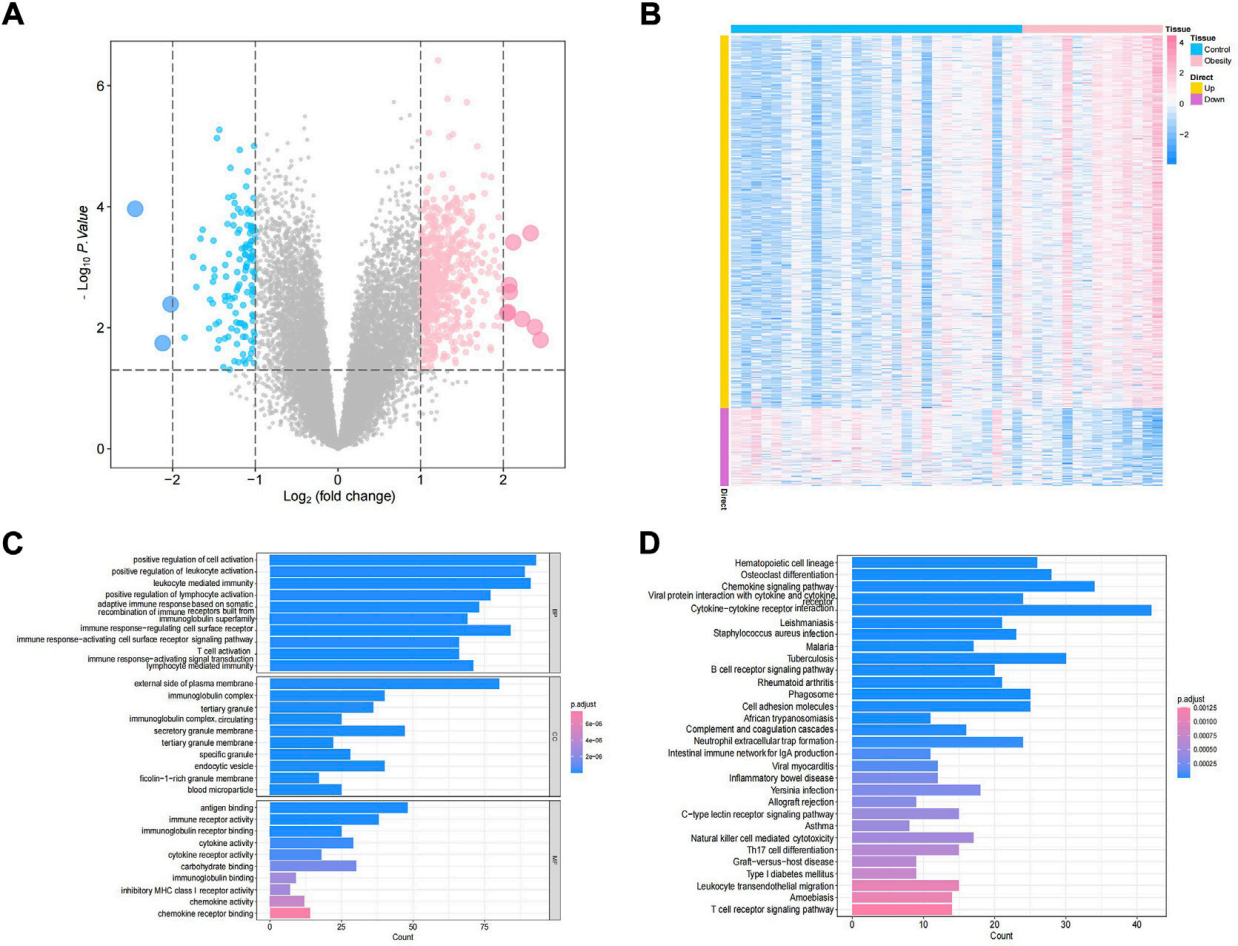
Identification of obesity differential genes. **(A)** Volcano plot of differential gene expression. Blue indicates differentially expressed downregulated genes, and pink indicates differentially expressed upregulated genes. **(B)** Differential gene expression heatmap, where blue indicates low-expression genes and pink indicates high-expression genes. **(C,D)** GO/KEGG enrichment pathway of differential genes.

**FIGURE 3:**
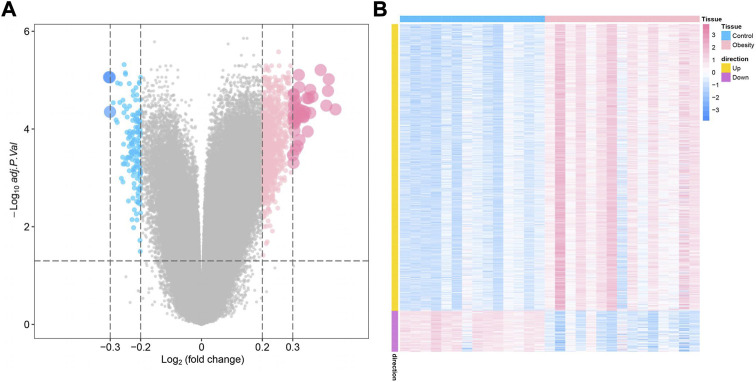
Identification of obesity differentially methylated genes. **(A)** Volcano plot of differentially methylated genes, where blue indicates differentially methylated downregulated genes and pink indicates differentially methylated upregulated genes. **(B)** Differential methylation heatmap, where blue indicates hypomethylated genes and pink indicates hypermethylated genes.

The study design of this research is displayed in Figure 1. In this study, GSE174475, including 43 cases of adipose tissue transcriptome data, was downloaded from the NCBI GEO public database. According to the expression profile data of adipose tissue, differential analysis was conducted to screen the differential genes between the control and obesity groups. A total of 742 differential genes were selected, including 614 upregulated genes and 128 downregulated genes (Figures 2A,B). We further performed pathway enrichment analysis on these differential genes. The Gene Ontology (GO) results showed that the differential genes were mainly enriched in the signaling pathways such as leucocyte-mediated immunity, adaptive immune response based on somatic, T cell activation, and lymphocyte-mediated immunity (Figure2C). The Kyoto Encyclopedia of Genes and Genomes (KEGG) results displayed that the differential genes were mainly enriched in the chemical signaling pathway, natural killer cell-mediated cytotoxicity, Th17 cell differentiation, and other signaling pathways (Figure 2D). On the other hand, we downloaded the 450-k data of GSE67024 from the NCBI GEO public database, including the methylation gene profiles in adipose tissue of 29 cases, and analyzed the differential methylation sites with the ChAMP package. A total of 1186 significant differentially methylated probes were screened, including corresponding 81 hypomethylated genes and 647 hypermethylated genes (Figures 3A,B).

### Combined analysis of DNA methylation and transcriptome in adipose tissue

**FIGURE 4:**
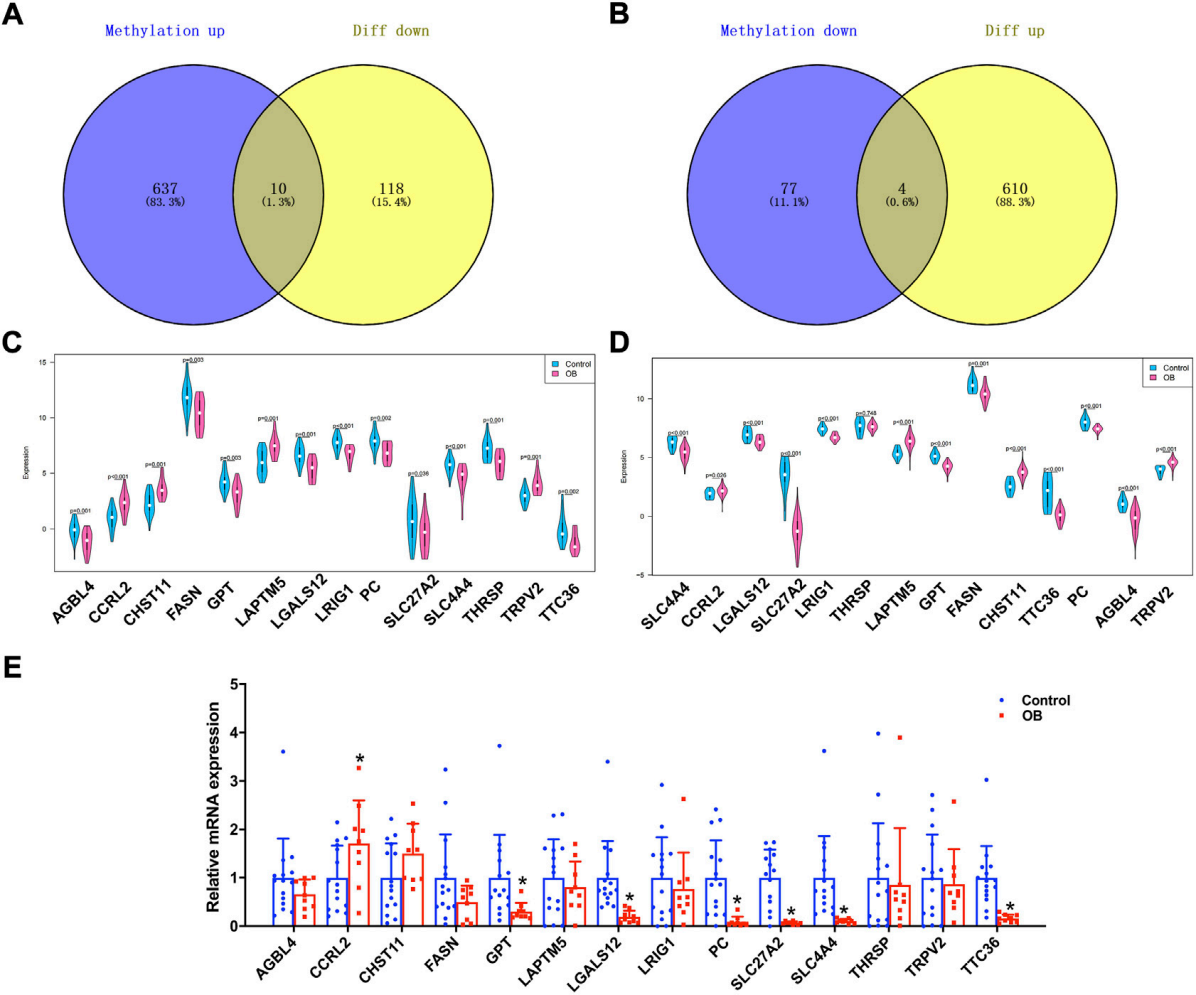
Identification of obesity hub genes. **(A,B)** Venn diagram of hypermethylated-low expression and hypomethylated-high expression genes. **(C,D)** Expression patterns of genes in GSE174475 and GSE156906. **(E)** qPCR experiments verified the expression of 14 genes.

Genes that were lower expressed in transcriptome analysis while being hypermethylated in the methylation dataset or higher expression genes with hypomethylation were further selected. The Venn plots showed that 14 genes were identified, namely, AGBL carboxypeptidase 4 (*AGBL4*), CC-chemokine receptor-like 2 (*CCRL2*), carbohydrate sulfotransferase 11 (*CHST11*), fatty acid synthase (*FASN*), glutamic-pyruvic transaminase (*GPT*), lysosomal protein transmembrane 5 (*LAPTM5*), galectin-12 (*LGALS12*), leucine-rich repeats and immunoglobulin-like domains 1 (*LRIG1*), pyruvate carboxylase (*PC*), solute carrier family 27 member 2 (*SLC27A2*), solute carrier family 4 member 4 (SLC4A4), thyroid hormone responsive (THRSP), transient receptor potential cation channel subfamily V member 2 (*TRPV2*), and tetratricopeptide repeat domain 36 (*TTC36*) (Figures 4A,B). Additionally, the present study verified the expression pattern of these genes through another adipose tissue dataset, GSE156906. The results showed that 13 of the 14 genes had the same expression pattern, namely, *AGBL4, CCRL2, CHST11, FASN, GPT, LAPTM5, LGALS12, LRIG1, PC, SLC27A2, SLC4A4, TRPV2*, and *TTC36* (Figures 4C,D). Moreover, we also verified the expression of key genes in adipose tissue between nine obese and 15 non-obese patients by RT-qPCR and found that seven genes remained in the same expression pattern in obese patients, namely, *CCRL2*, *GPT*, *LGALS12*, *PC*, *SLC27A2*, *SLC4A4*, and *TTC36* (all *p* value < 0.05) (Figure 4E). Therefore, these seven genes were regarded as potential key genes in obesity, and further analysis in exploring their potential molecular mechanisms in adipose tissue was conducted.

### Analysis of the immune microenvironment in adipose tissue

**FIGURE 5:**
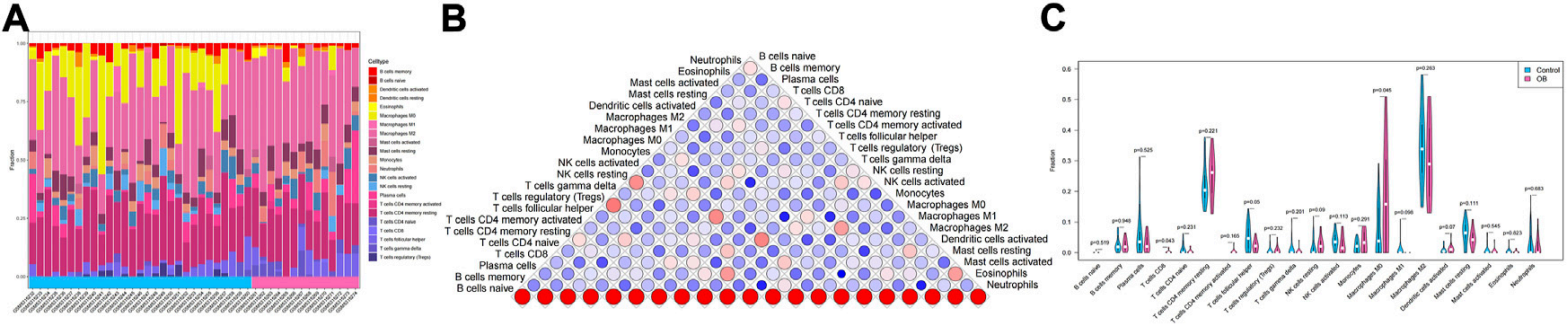
Immune infiltration of adipose tissue. **(A)** Relative percentage of 22 immune cell subpopulations in the sample. **(B)** Correlation among 22 immune cells, where blue indicates positive correlation and red indicates positive correlation. **(C)** Difference in immune cell content between normal samples and obese samples, with blue indicating normal samples and pink indicating obese samples. *p* < 0.05 was considered statistically significant.

**FIGURE 6:**
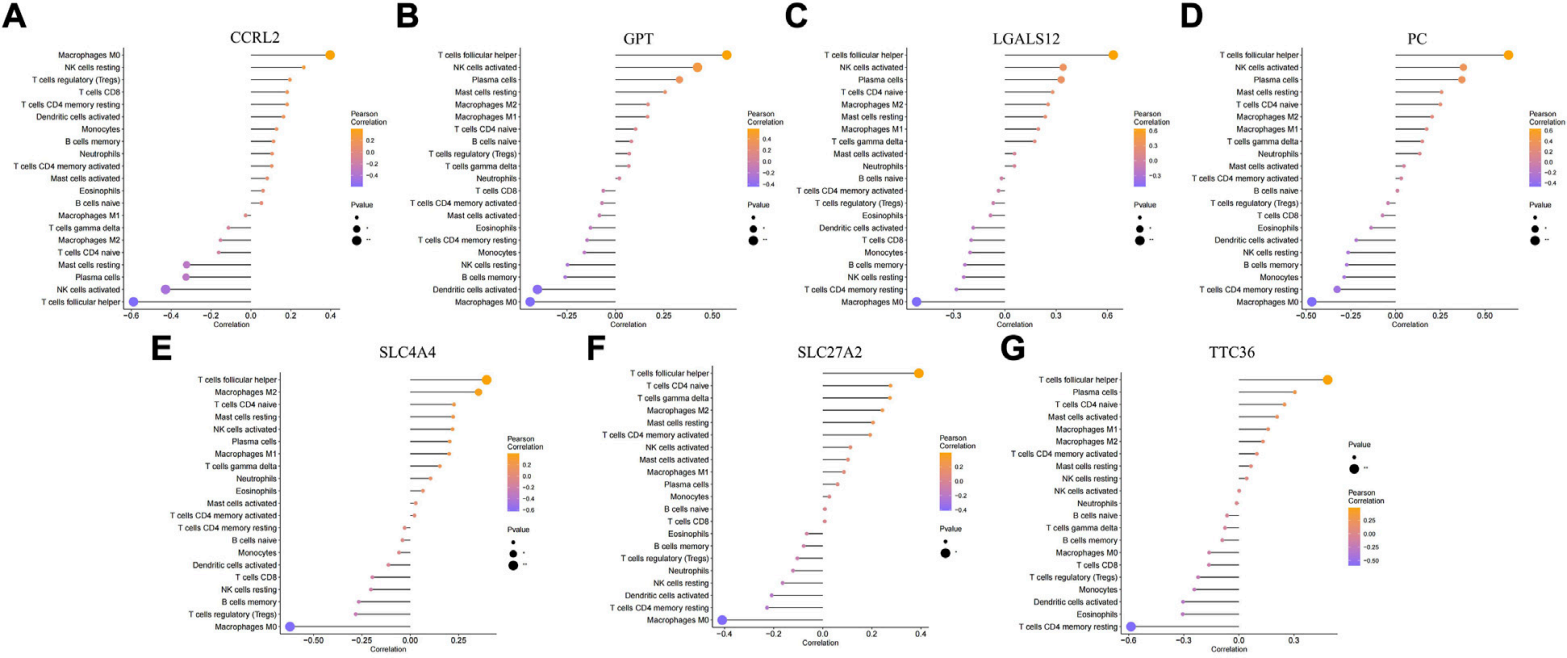
Correlation between key genes and immune cells. **(A–G)** Relationship between key genes and immune cells, and the genes are *CCRL2, GPT, LGALS12, PC, SLC4A4, SLC27A2,* and *TTC36*, respectively.

In addition to storing nutrients, adipose tissue is also an important immune organ in humans. It contains many immune cells, such as T cells, B cells, and macrophages. Under different microenvironments or stimulation, adipose tissue can secrete cytokines to recruit immune cells and further secrete various inflammatory factors, ultimately leading to chronic inflammation and insulin resistance. This study further explored the immune microenvironment of adipose tissue and the correlation between key genes and immune cell content in obesity based on the dataset GSE174475. We first quantified the content of immune cells in each sample by the CIBERSORT algorithm (Figures 5A,B). The results showed that the content of M0 macrophages was significantly increased in the obese group compared to that of the control group, while the content of T follicular helper cells was significantly decreased in the obese group (Figure 5C). The immuno-microenvironmental analysis based on GSE156906 also showed that the number of M0 macrophages was significantly increased in the obese group (Supplementary Figures S1A–C), which was consistent with the abovementioned results. Furthermore, we analyzed the correlation between the expression of key genes and the content of immune cells. The results showed that the seven genes were strongly correlated with the content of immune cells, suggesting that the key genes might affect the occurrence and development of obesity by influencing the immune microenvironment of adipose tissue (Figures 6A–G).

### Metabolic activity analysis of adipose tissue

**FIGURE 7:**
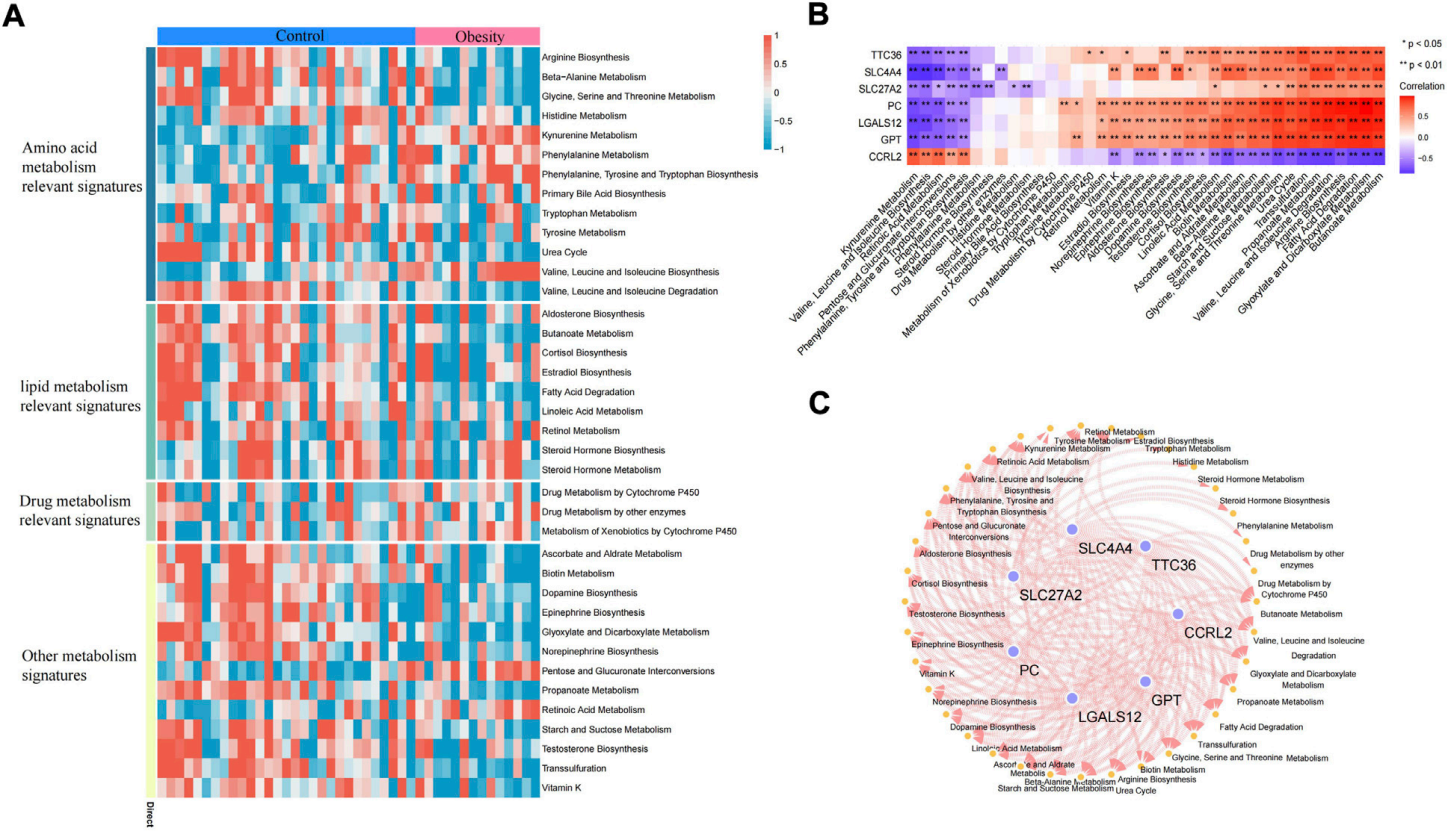
Metabolic pathway of adipose tissue. **(A)** Heatmap of the metabolic pathway of adipose tissue. **(B)** Correlation analysis between key genes and metabolic pathways. **(C)** Relationship between key genes and metabolic pathways (the correlation coefficient is greater than 0.3).

Adipose tissue can sense the energy metabolism of the body and then secrete adipokines to regulate energy homeostasis. The dysfunction of adipose tissue is manifested by the aggravation of inflammatory factors and insulin resistance. The dysfunction of adipose tissue and dysregulated glucolipid metabolism accelerate each other. In this study, the metabolic pathway score of each sample was quantified by the ssGSEA algorithm. In the metabolic pathway heatmap, significant difference in scores for lipid metabolism-related signatures and other metabolism signatures between the control and obese samples was observed (Figure 7A). Additionally, we also further quantified the correlation between key genes and metabolic pathways and constructed a key gene–metabolic pathway network (Figures 7B,C).

### Prediction of potential therapeutic drugs for obese patients

**FIGURE 8:**
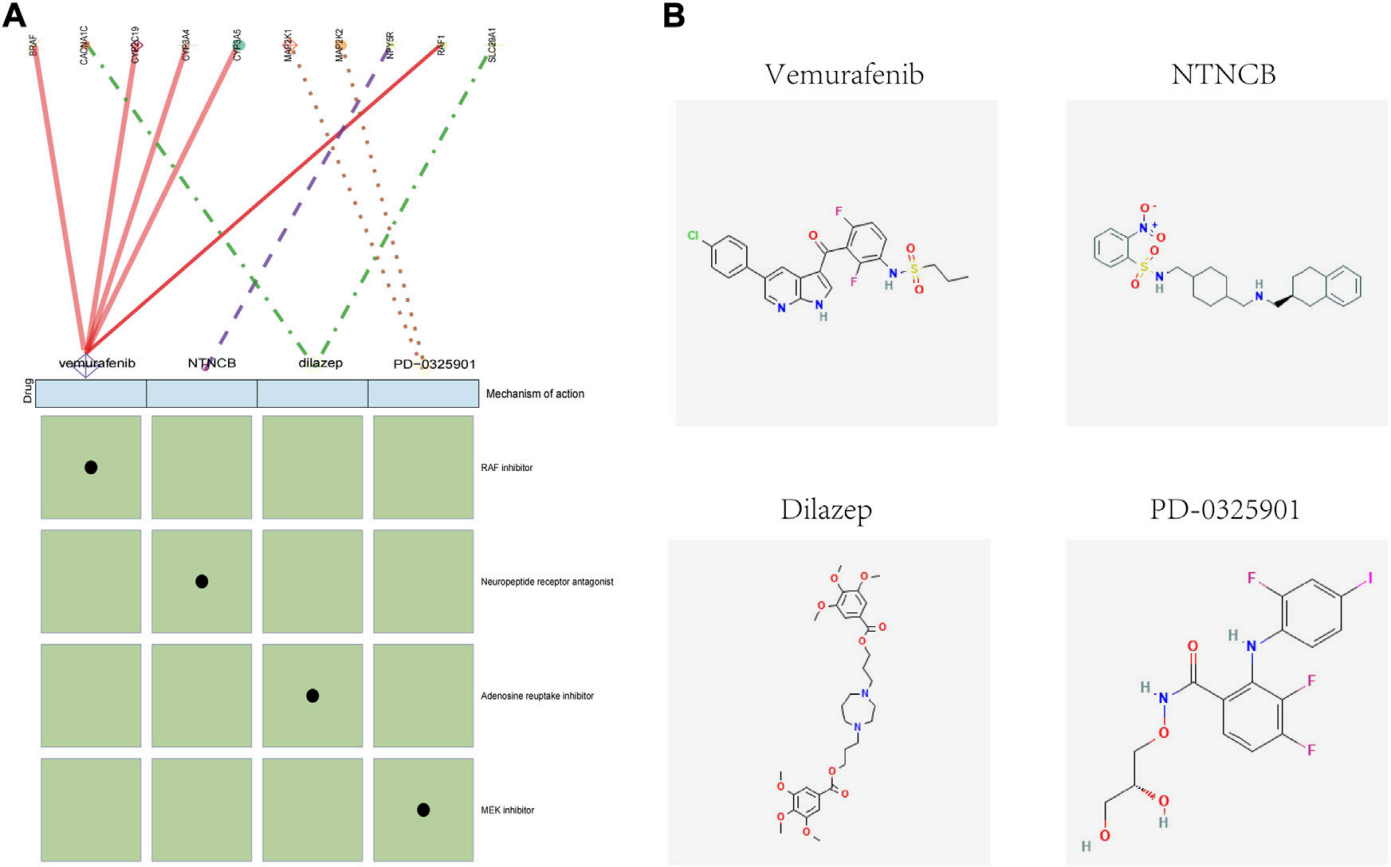
CMap analysis of the therapeutic drugs for obesity. **(A)** Molecular action and targets of potentially therapeutic drugs. **(B)** 2D visualization of these four drugs.

To identify potential drugs for the targeted treatment of obesity, we used the CMap database to evaluate the potential of small-molecule drugs in obesity treatment. We uploaded differentially expressed genes of adipose tissue to the CMap database and identified drugs related to obesity treatment. Among them, vemurafenib, NTNCB, dilazep, and PD-0325901 ((with available structure in PubChem database) were highly negatively correlated with obesity progression, indicating that these compounds might have therapeutic effects on obesity (Figure 8A). The mechanism of action (MOA) and drug targets of these drugs are identified using the CMap database to explore the potential mechanism of their treatment of obesity (Figure 8A), and the 2D visualization of these four drugs is shown (Figure 8B).

## Discussion

In this study, by analyzing three GEO datasets and verifying in 24 patients, we identified that *CCRL2, GPT, LGALS12, PC, SLC27A2*, *SLC4A4*, and *TTC36* might be the key genes that play an important role in obesity pathogenesis. Furthermore, these seven genes showed association with immune microenvironment and metabolic pathways in adipose tissue of obese patients. Additionally, we found that, compared to the control, M0 macrophage content was higher, while T follicular helper cell content was lower in adipose tissues in the obese group. Also, possible therapeutic drugs for obese patients were shown in our study. This study provided potential obesity-related therapeutic targets for further research and obesity management in clinical practice.

An increasing number of studies have shown that methylated genes are closely related to obesity. Simone et al. have identified 278 BMI-associated CpG sites in blood from epigenomic associations in European and Indian Asian populations, of which 120 CpG sites were consistently associated with BMI in both adipose tissue and blood. The epigenome-wide association study (EWAS) in the Asian population has also identified 116 BMI-associated CpGs, of which 108 were consistent with those of previous studies. In addition to being biomarkers, methylated genes have also been useful for predicting obesity and its related metabolic diseases (Wahl et al., 2017; Gallardo-Escribano et al., 2020). However, although many studies have suggested that excessive accumulation of fat caused most of the methylation changes (Wahl et al., 2017; Chen et al., 2021), others have insisted that methylation might be the cause of obesity (Mendelson et al., 2017). Additionally, methylated genes might also be associated with inflammation and lipoprotein-related biological pathways, further aggravating metabolic disorders (Wahl et al., 2017; Chen et al., 2021). These findings still need more experimental studies for further confirmation. In this study, seven candidate obesity-related methylation genes were identified, and our results suggested that these genes might participate in immune infiltration and in glucolipid metabolism.

Adipose tissue is also an important immune organ, containing many immune cells that function to maintain immune homeostasis. Infiltration and activation of immune cells during obesity is a powerful mechanism for adipose tissue remodeling (Engin, 2017), highlighting their potential as immunotherapeutic targets for the prevention and treatment of metabolic diseases. Studies in humans and mice have shown that the content of macrophages in adipose tissue is proportional to BMI (Weisberg et al., 2003). With obesity, adipose tissue is in a low-intensity inflammatory state, in which the percentage of macrophage infiltration in adipose tissue is significantly increased to 41% (Weisberg et al., 2003) compared with the normal state, which is consistent with the results of our study in terms that macrophages were significantly increased in the tissue of obese patients. More importantly, our analysis found that T follicular helper cells were significantly decreased in obesity, which has been rarely reported in previous studies. No reports have been found on the correlation between T follicular helper cells and adipose tissue or obesity. It should be noted that the BCL6 transcription repressor (Bcl6) has been shown to play an important role in regulating lipid metabolism in adipose tissue. Mice lacking Bcl6 showed multiple characteristics of lipid metabolism disorders, accompanied by adipose tissue dysplasia (LaPensee et al., 2014; Hu et al., 2016). Bcl6 is essential for the differentiation and development of T follicular helper cells (Yu et al., 2009), suggesting that the decrease in T follicular helper cells might be involved in the functional imbalance of adipose tissue in obesity.

In this study, consistent with previous studies (Xu et al., 2022), we found that *CCRL2* was significantly overexpressed in the subcutaneous tissues of obese subjects. However, this result needs to be interpreted with more caution, as one study suggested that the high expression of *CCRL2* in adipose tissue might be a compensatory result. Moreover, *CCRL2* lacks a signal transduction structure to transmit ligand signals into cells; hence, it might affect macrophage infiltration by competitively binding ligands (Xu et al., 2022). *GPT*, its protein that catalyzes reversible reactions of alanine and 2-keto glutaric acid, plays a key role in intermediate metabolism between glucose and amino acid metabolism. Furthermore, enzyme activity in the serum is usually used as a biomarker of liver injury, but it seems to be rarely investigated in the adipose tissue. Our result demonstrated the potential of *GPT* for subsequent basic research on glucolipid metabolism. Although some studies have shown that *LGALS12* was involved in blood glucose improvement (Corbi et al., 2017), many studies have revealed that it promoted adipose differentiation and development (Yang et al., 2004; Wu et al., 2018). Additionally, *LGALS12* knockdown accelerates lipid catabolism (Baum, 2011; Yang et al., 2011). These results suggest that *LGALS12* acts as a negative regulator in obesity, which is inconsistent with our results, and this inconsistency might be explained by the possibility of adaptive regulation. As a human adipocyte-specific lncRNA, *ADIPINT* regulates lipid metabolism in adipocytes by regulating PC protein abundance and enzymatic activity (Kerr et al., 2022). Because the effects of *PC* often correlate with its activity, the reduced transcript levels of *PC* found in obese individuals in this study were not fully representative of its functional characteristics. Some studies have shown that *SLC4A4* may be involved in the pathogenesis of obesity and also participate in immune infiltration (Li et al., 2015; Li et al., 2022), and the methylation of the solute carrier protein (*SLC*) gene has been closely related to BMI and waist circumference (Mendelson et al., 2017; Sayols-Baixeras et al., 2017). In this study, not only *SLC4A4* but also *SLC27A2* were both significantly downregulated in obese patients, which to a certain extent supports previous studies and suggests that these two genes might be key genes requiring more attention in the future exploration of the obesity mechanism. Although there are no relevant reports on obesity or adipose tissue for *TTC36*, few studies have found that *TTC36* was negatively regulated by *TTC36* methylation in liver cancer tissues (Jing et al., 2022), and it is involved in the process of immune infiltration. It suggests that the role of *TTC36* in adipose tissue might be worthy of further investigation, especially in immune infiltration. In short, there are currently limited reports about these seven key genes, and more experimental research and clinical trials are needed. However, these genes may serve as potential target genes for subsequent basic research.

Our results showed that vemurafenib, NTNCB, dilazep, and PD-0325901 might have therapeutic effects on obesity. Unfortunately, for now, the direct effect of these drugs on obesity was hardly reported. Interestingly, according to the molecular action of potential therapeutic drugs analysis, *SLC29A* was indicated as a potential target in the possible mechanism of dilazep in treating obesity. Recent research suggested *SLC29A* was considered to regulate inosine levels in brown adipose tissue and consequently enhanced thermogenesis (Niemann et al., 2022); hence, these may be the indirect evidence supporting dilazep as the potential therapeutic drug. However, caution is needed until more evidence on the anti-obesity of these drugs is found.

In summary, we successfully identified seven key genes, namely, *CCRL2*, *GPT*, *LGALS12*, *PC*, *SLC27A2*, *SLC4A4*, and *TTC36*, which are involved in obesity occurrence and development likely through the immune microenvironment. Meanwhile, M0 macrophage content was higher, while T follicular helper cell content was lower in adipose tissues in the obese group. Our results also indicated that vemurafenib, NTNCB, dilazep, and PD-0325901 might have therapeutic effects on obesity.

## Data Availability

The datasets presented in this study can be found in online repositories. The names of the repository/repositories and accession number(s) can be found below: https://www.ncbi.nlm.nih.gov/geo/, GSE67024, https://www.ncbi.nlm.nih.gov/geo/, GSE174475, https://www.ncbi.nlm.nih.gov/geo/, GSE156906.
