# Supplementary material for: Comprehensive analysis of epigenomics and transcriptome data to identify potential target genes associated with obesity
Source: Front Genet. 2022 Oct 14;13:1024300. doi: 10.3389/fgene.2022.1024300 (PMC9614047; doi:10.3389/fgene.2022.1024300)
Supplement: Supplementary file 1 [file DataSheet1.zip › supplementary materials/Supplementary File 2.docx]

Table 1. Basic demographic characteristics of enrolled datasets

|  | Age | Female/Male | BMI | |
| --- | --- | --- | --- | --- |
|  |  |  | Control | Obese |
| GSE67024 | 45.24±11.14 | 29/0 | 25.11±2.49 | 41.36±4.53 |
| GSE174475 | 49.40±9.80 | 43/0 | 26.45±2.47 | 34.31±3.17 |
| GSE156906^[1]^ | 38.00±1.00 | 56/14 | 22.90±0.40 | 38.45±0.90 |

Table 2. Comparison between Control and Obese in dataset GSE67024

|  | Control  (n=14) | Obese  (n=15) | p |
| --- | --- | --- | --- |
| Age | 44.64±11.86 | 45.80±10.82 | 0.786 |
| BMI | 25.11±2.49 | 41.36±4.54 | ＜0.001 |

Table 3. Comparison between Control and Obese in dataset GSE174475

|  | Control  (n=29) | Obese  (n=14) | p |
| --- | --- | --- | --- |
| Age | 48.93±9.43 | 50.36±10.85 | 0.660 |
| BMI | 26.45±2.47 | 34.31±3.17 | ＜0.001 |

Table 4. Comparison between Control and Obese of enrolled patients

|  | Control  (n=15) | Obese  (n=9) | p |
| --- | --- | --- | --- |
| Male/Female | 7/8 | 7/2 | 0.210 |
| Age | 47.33±10.66 | 50.89±8.92 | 0.411 |
| BMI | 22.43±2.81 | 30.01±1.48 | ＜0.001 |

**References**

[1] A. Fuchs, D. Samovski, G.I. Smith, et al., Associations Among Adipose Tissue Immunology, Inflammation, Exosomes and Insulin Sensitivity in People With Obesity and Nonalcoholic Fatty Liver Disease, Gastroenterology 161(3) (2021) 968-981 e12.
