# Supplementary figures and images for: Comprehensive analysis of epigenomics and transcriptome data to identify potential target genes associated with obesity
Source: Front Genet. 2022 Oct 14;13:1024300. doi: 10.3389/fgene.2022.1024300 (PMC9614047; doi:10.3389/fgene.2022.1024300)

A

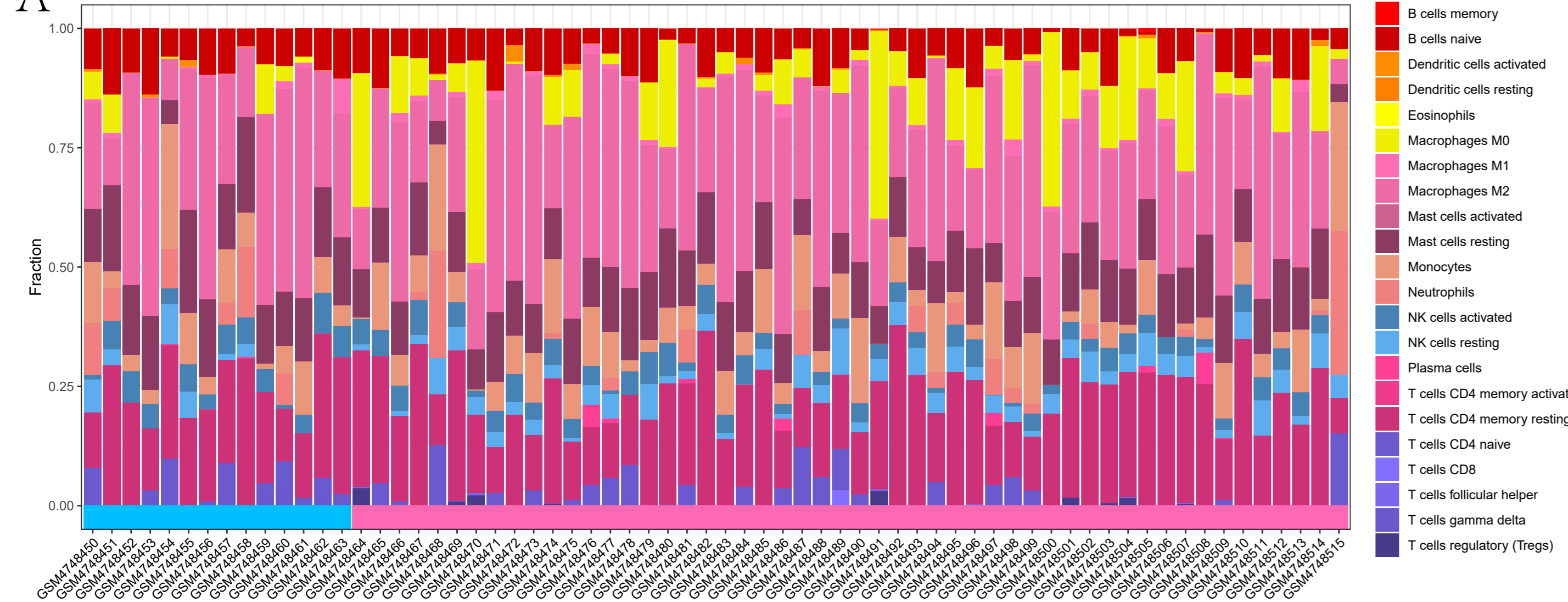

B

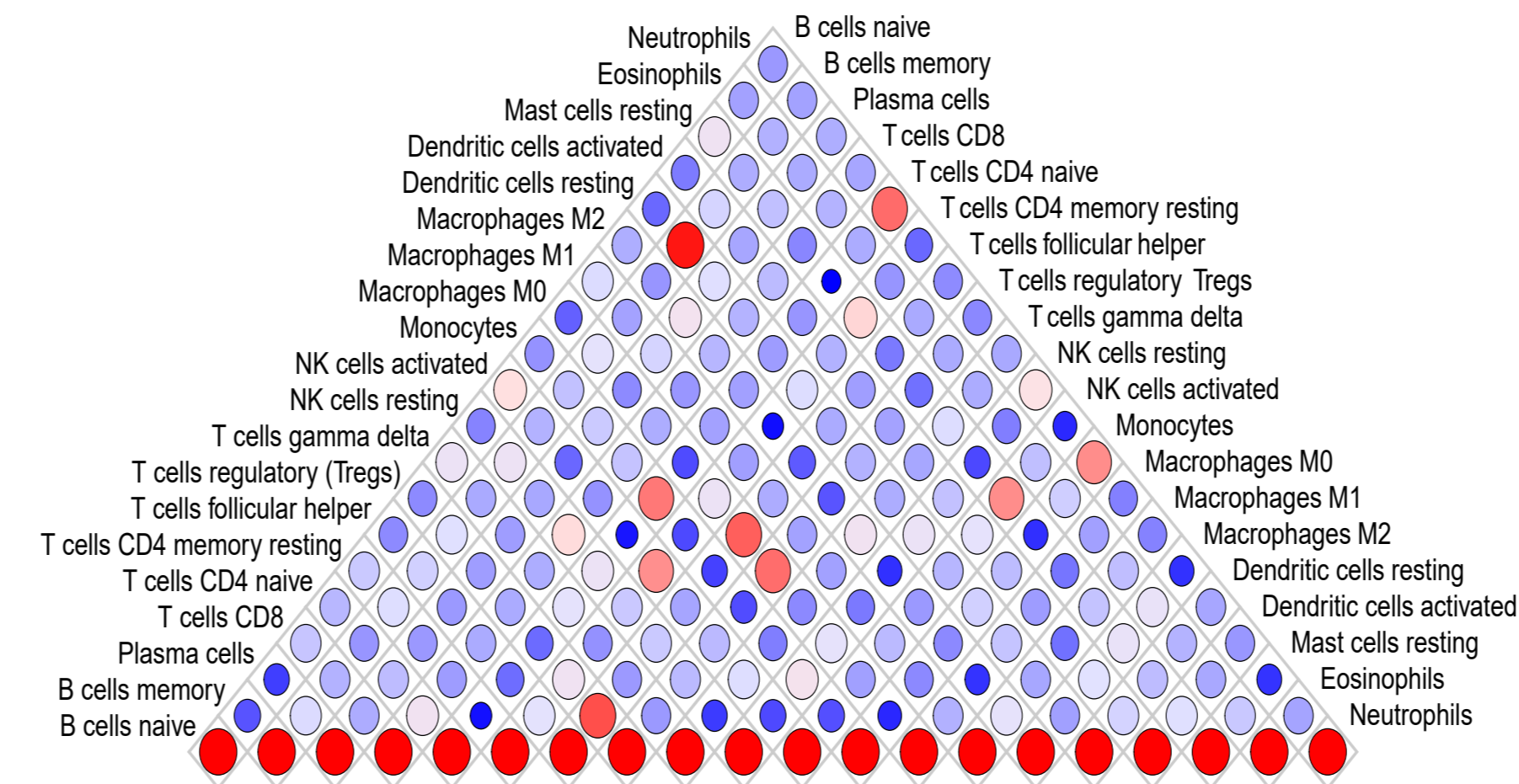

C

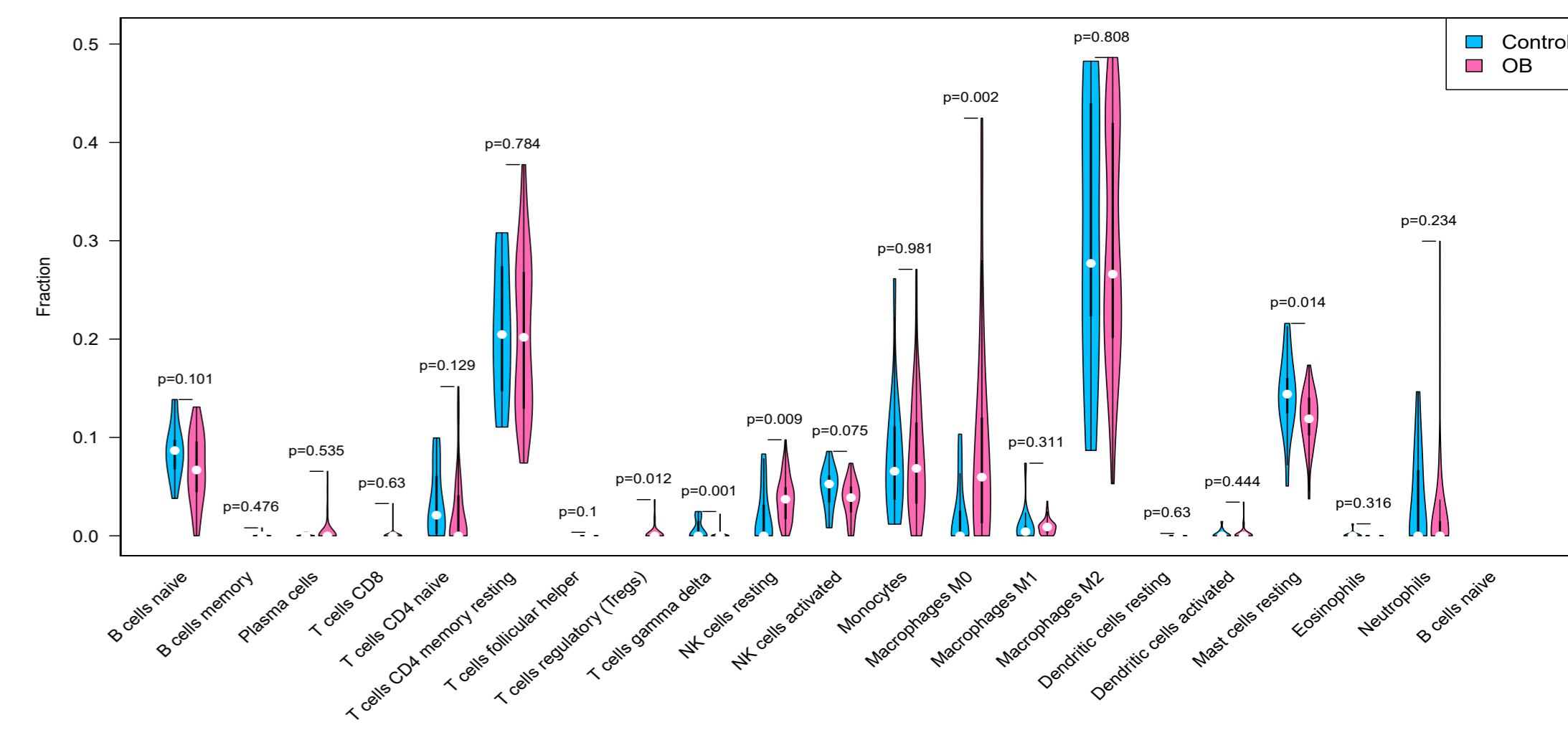

Supplement: Supplementary file 1 [file DataSheet1.zip › supplementary materials/Supplementary File 3.PDF]
